# Effect on *Ziziphus jujuba* Mill. fruit powders embedded on physicochemical properties, biological activities, and rheologic quality of cake

**DOI:** 10.1002/fsn3.3276

**Published:** 2023-03-09

**Authors:** Meriem Elaloui, Youssef Ammari, Hanen Ghazghazi, Afwa Ep Ben Othmen Gorrab, Abdelwahed Laamouri, Rim Driouich Chaouachi

**Affiliations:** ^1^ National Institute of Research in Rural Energining Water and Forests (INRGREF) Carthage University Ariana Tunisia; ^2^ Higher Institute of Biotechnology Sidi Thabet Manouba University Ariana Tunisie

**Keywords:** bioactive compounds, physicochemical analysis, rheological characteristics, sensory analysis, *Ziziphus jujuba* fruit

## Abstract

This study aimed to improve the effects on the physical, textural, and rheological characteristics of cake supplied with *Ziziphus jujuba* fruit powder at rates of 0%, 3%, 5%, and 10%. The Physicochemical and antioxidant as well as the antibacterial activity and sensory qualities of *Z. jujuba* fruits were also investigated. The highest levels reached the values of 245.15 mg GAE/g DW (phenol) and 180.23 mg RE/g DW (flavonoids). Pulp extracts were also subjected to HPLC analysis in order to identify and quantify the sugar composition. This technique allowed us to identify Mahdia as the richest provenance especially in glucose (136.51%) and sucrose (113.28%) contents. The antioxidant activities investigated using DPPH assay decreased slightly from 175 μg/mL (Sfax) to 55 μg/mL (Mahdia). Furthermore, the antibacterial activity indicated that the *S. aureus* was the most inhibited especially by Sfax powder extracts (from 12 to 20 mm). Our results showed that the incorporation of *Z. jujuba* powder ameliorated the physicochemical and rheological characteristics (humidity, gluten yield, tenacity, falling time, and configuration) of the dough. Sensory analysis showed that consumer scores were increased with the increasing supplementation powder levels. Highest scores attributed to the cake supplied with 3% jujube powder collected from Mahdia provenance and confirmed that *Ziziphus* fruit could be advised a part of our diet. These results could validate a novel method to conserve *Z. jujuba* fruits in order to avoid their soilages for a long period.

## Graphical abstract

*Ziziphus jujuba* fruits could be an active ingredient in the food industry by transforming it to cake and increase the consummation of *Z. jujuba* fruit that could solve the problem of conservation for long periods.

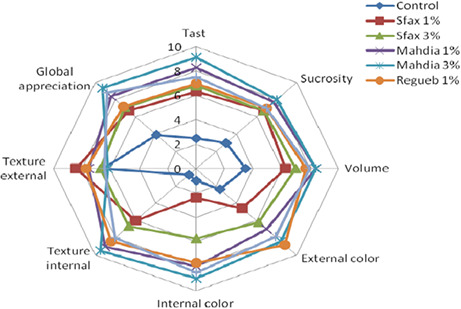

## 1 INTRODUCTION

*Ziziphus jujuba* fruit, known as Anneb in Tunisia, is an upright tree with bright green, glabrous leaves (Elaloui et al., 2014). In summer, especially from September to mid‐October, the period of full maturity, this species offers an obovoid fruit having 1.7 cm for length and 1 cm of width with a large range of color from brownish yellow to dark brown. It was not only tastier than apple but also similar to date fruit. So why it was named Chinese jujube or Chinese date. *Ziziphus* pulps were very famous for their richness in mineral fibers, vitamins, and polyphenols (Elaloui et al., 2020; Rashwan et al., 2020). Unfortunately, shelf life could not exceed more than 3 months because jujube cannot be stored for a long period under ambient conditions. In addition, some properties such as vitamin C and antioxidant activity cease in fruit stored in cold conditions. Many problems were associated with jujube storage. Tunisian people usually store the fruit at −5°C. In fact, this fruit can be freeze‐dried or frozen. Many methods were used to dry *Z. jujuba* fruits such as sun oven, microwave, and freeze‐drying, but the most to conserve jujube was the microwave. Other's studies showed that the sun dry method was the efficient technical to dry *pinus halepensis* Mill. seeds with the highest (0.08 and 0.05 mg/mL) antioxidant activities (Mahdhi et al., 2021).

So in order to increase the consumption of jujube and to conserve it for a longer duration, we tried to use this fruit as an active ingredient in the food industries by transforming it to powder and adding it to jam, cookie, and cake (Rashwan et al., 2020). The idea to combine *Z. jujuba* fruit powder in food products, such as cake, could play an important role in stored pulp protection and reduction of the associated risks. In fact, food companies were faced with an increased demand by health professionals and consumers for healthier food products. In addition, this combination contributed to the nutritional and therapeutic benefits by using this fruit as an active ingredient in the food industry.

However, to the best of our knowledge, no studies were reported on cake enrichment with *Z. jujuba* fruits. So this investigation aimed to establish the impact of this incorporation on the physicochemical and rheological quality of the flours and on the sensory quality of the *Z. jujuba* cake. The result revealed that jujube powder could be a better substitute for wheat flour in preparation of flour products. In addition, Tunisian *Z. jujuba* fruit was known for its long history of uses as a vital food and for traditional medicine (Rashwan et al., 2020). It could be used or consumed as beverage, juice, brandy, wine, jelly, and also candied jujube. In addition, the biochemical fruit composition's enhanced many therapeutic effects against several diseases such as obesity, cardiovascular disease, diabetes, and even certain types of cancers (Elaloui et al., 2016; Etebari et al., 2015). Therefore, it was the suitable time to improve the technological quality and nutritional value of this fruit. In this context, the aim of the present work was to evaluate biochemical *Z. jujuba* fruit powder contents collected from five Tunisian provenances (Choutrana, Mahdia, Regueb, Sfax, and Mahres) and to evaluate their effects on physicochemical properties and sensory evaluation of cake.

### 1.1 Plant material

**FIGURE 1:**
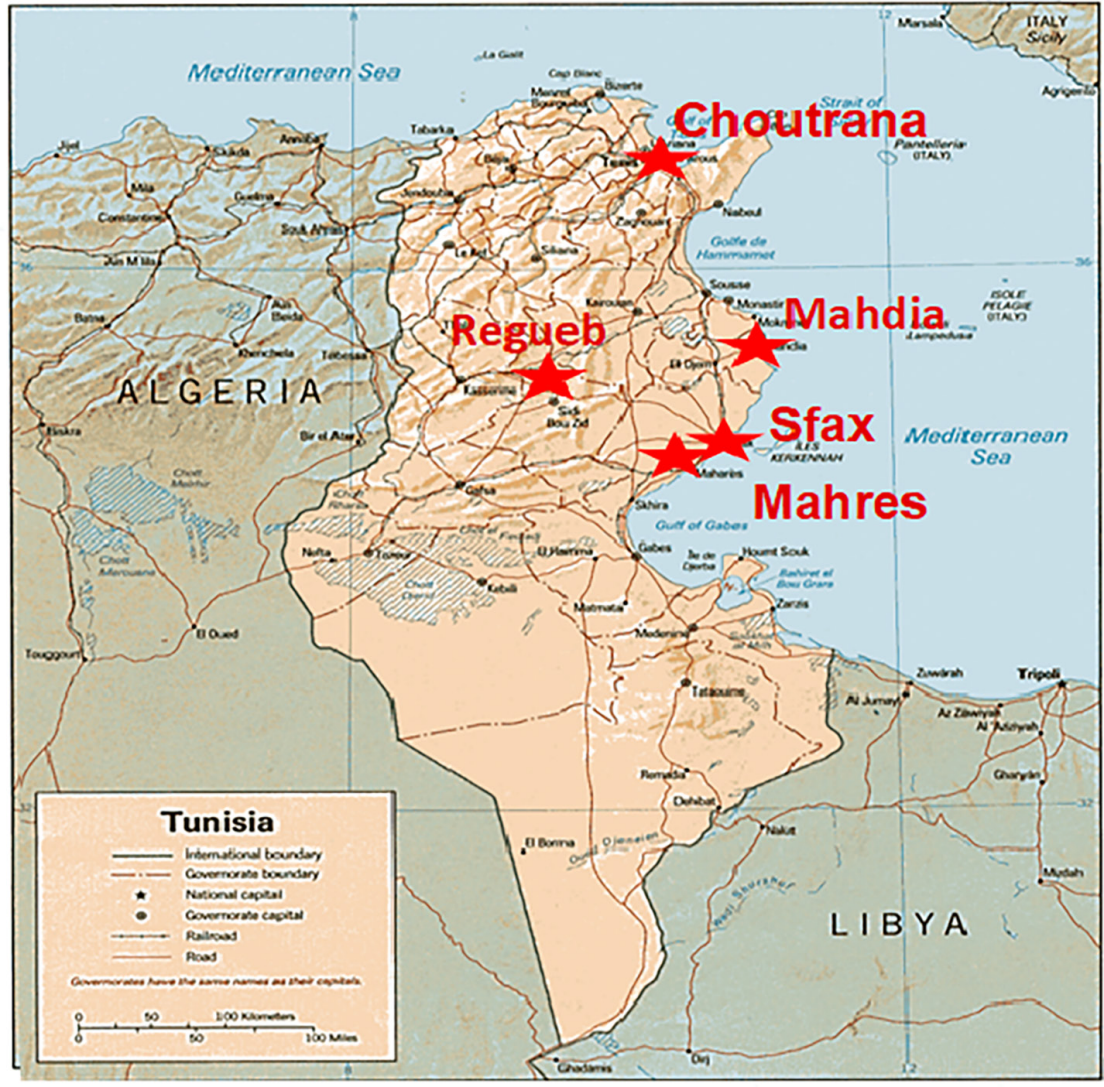
Geographic distribution of five *Ziziphus jujuba* provenances.

*Z. jujuba* fruits were collected in September 2020 (the period of jujube maturation in Tunisia) from the areas around Choutrana, Mahdia, Regueb, Sfax, and Mahres (Figure 1). Plant botanical identification was carried out by Professor Mohamed Boussaid and a voucher sample (ZJF2020) was deposited at the Herbario of the National Institute for Research in Rural Engineering, Water and Forests (INRGREF) in Tunisia. These provenances were planted in the Rouhia experimental station situated in northwestern Tunisia (35°40′–15.39″ N; longitude 9°0.3′–15.29″ E; latitude 636 m) under semi‐arid bioclimate.

This experimental station with an area of 200 km^2^ was characterized by irregular rainfall events especially between January and February (El Aloui, 2013). The temperature levels ranged from 4°C (January) to 33.4°C (July and August).

## 2 METHODS

The ripe jujube fruits (selection was based on reaching the stage of full maturity in terms of color, flavor, and structure) were immediately transferred to our laboratory. So the disease‐free and identical in shape jujubes were selected for the sun drying process in order to evaporate water from fruits by sun and conserve their biochemical and biological activities (Anjum et al., 2020). The dried fruits were first grounded using a mill equipped with a grid with holes 1.00 mm in diameter and then stored in plastic bags until the chemical analysis began.

### 2.1 Mineral composition

The mineral composition (K, Fe, Ca, and Mg) of *Z. jujuba* fruits was evaluated according to calcination methods. Briefly, 0.5 g of *Z. jujuba* fruit powder was calcinated at 550°C for 4 h. The ash, already cooled, was moistened with a few drops of distilled H_2_O (3 mL). It was combined with 10 mL of hydrochloric acid and then filtered into 50 mL graduated flasks. The solution was then stored in vials until it was analyzed. The mineral element contents were determined using a flame spectrophotometer (Genway, Model PSP7).

### 2.2 Physicochemical analysis of *Z. jujuba* fruits

Physicochemical analysis focused specifically on the water, fat, and protein contents. The water content was determined after desiccation of the fresh material at a temperature of 103 ± 2°C in an isothermal oven ventilated at atmospheric pressure to a nearly consistent measurement. The protein contents were evaluated according to Kjeldahl methods (1883). Water level contents were evaluated by weight difference after drying the fresh pulp (Audigie et al., 1978). *Z. jujuba* powder fat contents were achieved by extracting samples in a Soxhlet apparatus using the hexane as solvent. The extractions were carried out in duplicate.

### 2.3 Sugars extraction

#### 2.3.1 Yield extraction

*Ziziphus* fresh pulp (1 g) was macerated in 10 mL of Ultra High Quality (UHQ) water for 30 h. After centrifugation and filtration, the supernatant extracts were kept in the freezer before HPLC analysis.

#### 2.3.2 Sugar analysis by HPLC method

Sugars were analyzed in triplicate using HPLC Dionex (P680‐ASI 8100‐Agilent) equipped with a Carbo Pac PA1 column (250 × 4 mm). The postcolumn injection was used with a volume of 25 μL. The HPLC worked under suitable programs: First it was stabilized with 1 mM of KOH during 10 min, and then 100 mM KOH solution was used as a mobile phase during the analysis. Evaluation was done at a flow rate of 1 mL min^−1^with constant Helium purge. The identification and the quantification of the compounds were performed in comparison with the commercial standards. A column wash between two successive injections was made with methanol.

### 2.4 Secondary metabolite contents

The total phenol contents (TPC) were carried out by the Folin Ciocalteau's reagent (Singleton & Rossi, 1965). Results were expressed as mg of Gallic acid equivalents per gram of dry weight (GEA/g DW). Briefly 1 mL of extracts and of Folin Ciocalteu (1.25 mL) was added to 2 mL of sodium carbonate (7%). The absorbance was carried out at 765 nm using Shimadzu 1600‐ UV spectrophotometer. Flavonoid contents (FC) of jujube extracts were determined according to Earp et al. (1981) methods. So 1 mL of each extract was mixed with 1 mL of AlCl_3_ (2%) in darkness. The flavonoid was used to prepare the curve calibration and the absorbance was measured at 430 nm. Results were expressed as mg quercetin equivalent per gram dry weight (mg QE/g DW). All analyses were run in triplicate. Tannin condensed contents (TC), expressed as mg Catechin Equivalent per gram dray weight (mg CE/g DW), werealso quantified (Sun et al., 1998). A Shimadzu 1600‐UV spectrophotometer was used to perform the absorbance at 500 nm.

### 2.5 Biological activities

#### 2.5.1 Antioxidant activity

##### DPPH radical scavenging activity

The antioxidant activity was investigated according to Ben Hmed et al. (2020) method with some modifications. Therefore, different concentrations of pulp extracts were prepared and incubated (V/V) in darkness conditions for 30 minutes. The absorbance was read at 734 nm after 30 min of incubation at 25°C after curve calibration with standard or DPPH solution (2.36 mg in 100 mL of ethanol). Tests were carried out in triplicate and the IC_50_ (Concentration required for 50% inhibition) was determined according to the following formula

$$\text{Scavenging effect}\;\left(\%\right)=\left[\left(A_{0}-A_{1}\right)/A_{0}\right]\times 100.$$

where a_0_ is the absorbance of the blank sample and a_1_ is the absorbance in the presence of the sample.

#### 2.5.2 Antibacterial activities

The antibacterial activities were evaluated using 2 g‐positive bacteria: *Staphylococcus aureus* ATCC 25923 and *Listeria monocytogenes* ATCC 070101121 and 2 g‐negative bacteria: *Kleibsella Pneumoniae* ESA 8 and *Aeromonas hydrophila*.

These species were provided by the Veterinary Epidemiology and Microbiology, Bacteriology and Development Groups Biotechnology Laboratory (Tunisian Institute of Pasteur).

##### Agar diffusion method

The various methanol extracts from *Z. jujuba* were dissolved in sterile water. Before use and for the detection of the antimicrobial activity, each extract was diluted to 50 μg/mL and sterilized by filtration through a 0.2 μm pore size filter.

Antibacterial tests were performed by agar well diffusion methods as described by Dharajiy et al. (2016). Broth microdilution assay using sterile Mueller‐Hinton media (BioRad, France) for bacterial strains and yeast malt extract agar YMA (Bio‐Rad, France) for antifungal tests were used. A fresh cell suspension (0.1 mL) adjusted to 10^7^ CFU/mL for bacteria and 10^5^ cell/mL for fungus were inoculated onto the surface of agar plates. Afterward, wells 6 mm diameter, were punched in the inoculated agar medium and 30 μL of the extract was added to each well. Negative controls consisted of using 30 μL water. The plate was allowed to stand for 40 min at 4°C to permit the extract diffusion followed by incubation at 37°C for 24 h for bacteria. The antibacterial activities were evaluated by measuring the zones of inhibition (clear zone around the well) against the test microorganisms. All tests were repeated three times (Essghaier et al., 2014).

##### Determination of minimum inhibitory concentration (MIC)

The minimum inhibitory concentration (MIC) of each extract was determined by using the microdilution broth method. MIC values were estimated visually by the absence of turbidity as previously reported by Khemiri et al. (2020).

### 2.6 Effects of adding jujube flour on the physicochemical and rheological characteristics of the cake

#### 2.6.1 Cake preparation

The cake was prepared using the Expert Design V 6 in order to formulate a product that meets customer and industrial approval.

Cakes were prepared according to Najjaa et al. (2020) methods. For each, we mixed 134 g sugar powder, 90 g eggs, 85 mL milk, 85 mL oil, 6.5 g vanilla, and 0.5 g salt. Samples were prepared in the same conditions (duration, mixing, temperature). Jujube powder was added in the ratios of 0% (control: F1), 3% (F2), 5% (F3), and 10% (F4). The cakes were prepared in a local pastry industry. The eggs were mixed with an electric mixer (Electra EK‐230 M, Japan) at 128 rpm for 4 min with sugar. The oil and the milk were added to mixture (M_1_). A second mixture (M_2_) was prepared by adding jujube powder or wheat flour with vanilla. Finally, the two mixtures were combined. The baking operation was then carried out in an oven (Zuccihelli Forni, Italy) at 180°C for duration of 30 min.

After cooling, each sample was packed in polyethylene bags for evaluation of different characteristics and stored at ambient temperature.

#### 2.6.2 Rheological characterization

**FIGURE 2:**
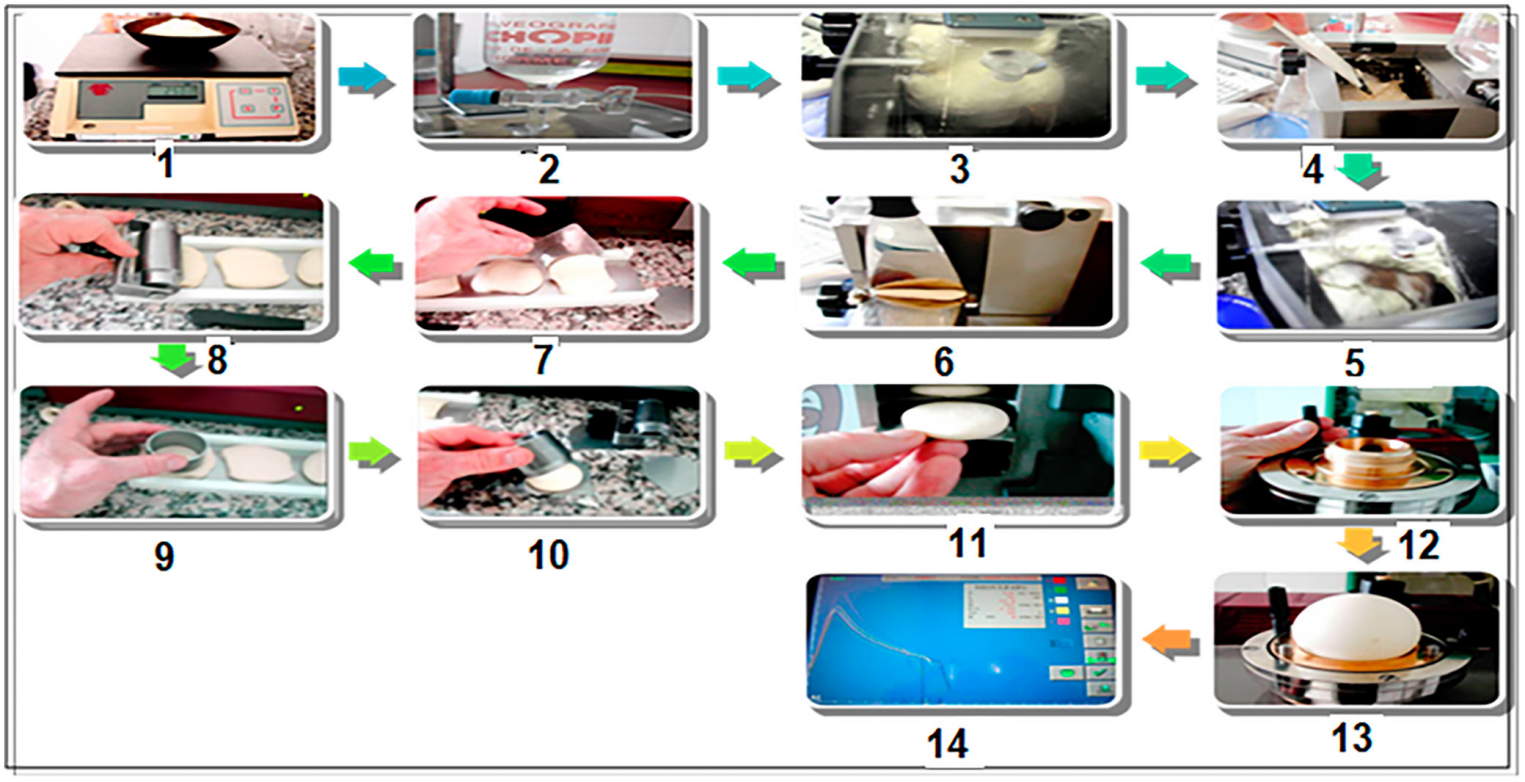
Different stages of the Chopin Alveograph test used to characterize *Ziziphus jujuba* dough.

The dough was characterized rheologically by a Chopin Alveograph (according to NF V03.710, 1991; ISO 5530‐4, 1991). This method was used to describe dough resistance against the biaxial deformation. Different stages of the Chopin Alveograph test were resumed in Figure 2.

#### 2.6.3 Sensory analysis

Sensory analysis defined as texture, flavor (taste), aroma, and visual aspect was achieved according to international standardized (NF ISO 5492) hedonic method (Drake et al., 2004). So sixty panelists aged between 20 and 60 years old were used to carry out this test. Results were noted according to a numbered scale from 0 (unacceptable) to 7 (very desirable). Each panelist noted his appreciation for cake supplemented by *Z. jujuba* fruits. The studied parameters were taste, color, texture, volume, and global appreciation.

Samples were presented in arbitrary order to each panelist on plastic plates using a 3‐digit code for each sample.

#### 2.6.4 Statistical analysis

Statistical analysis results were statistically analyzed using STATISTICA (Statsoft, 1998). Each data presented was mean ± standard deviation of three replicates. Multiple mean comparisons were performed using the Student–Newman Keuls test with a significance level of *p* = 0.05.

## 3 RESULTS

### 3.1 Fruit characterization

Jujube fruit was known for many uses as a vital food due to its richness on many components such as ash, protein, fat, and carbohydrate (Table 1). This composition was affected by the maturity stage of the fruit. Furthermore, others showed that the variation of these chemical compositions depended on the provenance.

**TABLE 1:** Chemical composition of *Ziziphus jujuba* fruits collected from five provenances.

Provenances contents	Sfax	Choutrana	Mahres	Mahdia	Rgueb
Water (%)	9.12 ± 0.03 D	9.7 ± 0.04 B	9 ± 0.02 E	10.1 ± 0.05A	9.42 ± 0.05C
Dray weight (%)	90.88 ± 0.03 B	90.3 ± 0.04D	91 ± 0.02A	89.9 ± 0.05 E	90.58 ± 0.05 C
Ash (%)	1.65 ± 0.017 C	1.79 ± 0.018B	1.56 ± 0.02D	1.85 ± 0.016A	1.67 ± 0.014C
Fat (%)	8.22 ± 0.13 E	9.76 ± 0.08C	8.97 ± 0.06 D	12.51 ± 0.15A	11.64 ± 0.11B
Protein levels (%)	0.45 ± 0.004 D	0.4 ± 0.008 E	0.64 ± 0.01 A	0.61 ± 0.006B	0.53 ± 0.009C
Calorific power (Kcal/100 g MF)	135.54 ± 1.11D	145.92 ± 0.8C	145.93 ± 0.58C	181.39 ± 1.38 A	171.16 ± 1.14B

*Note*: The data are mean values of three measurements. Confidence intervals were calculated at the threshold of 5%.

The analysis of the protein levels varied between 0.40% and 0.64%. The Mahres provenance was the richest one, and the Choutrana was the poorest one (Table 1).

The humidity levels varied between provenances. It ranged from 9% (Mahres) to 10.1% (Mahdia). This variability could be due to some conditions such as climatic conditions (temperature, precipitation…) and geographic location…. This low percentage facilitated the storage of *Z. jujuba* fruits.

Ash analysis varied between 1.71% DW and 2.05% DW for Mahdia and Mahres respectively. Such levels were more important than those (0.82% DW) for *Z. lotus* (Murdock, 2002).

### 3.2 Total sugar contents

**FIGURE 3:**
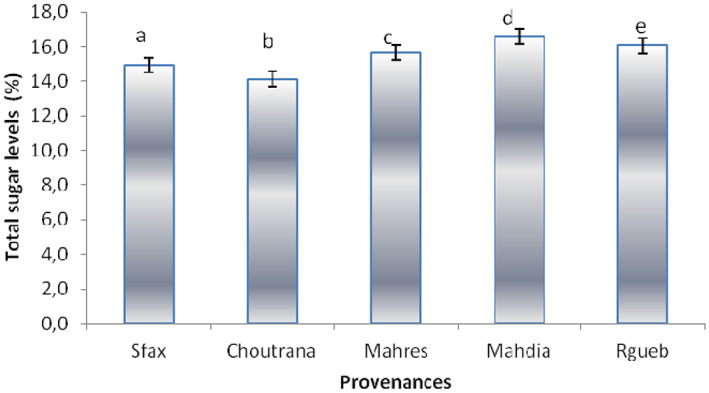
Total sugar yields (%) of *Ziziphus jujuba* fruits. The data are mean values of three measurements. Confidence intervals were calculated at the threshold of 5%.

Total sugar levels as mentioned in Figure 3 ranged from 14.12% to 16.59%. The Mahdia provenance was the richest one. The present results could enhance the nutritional value of *Z. jujuba* fruit.

#### 3.2.1 Sugar HPLC analysis

**FIGURE 4:**
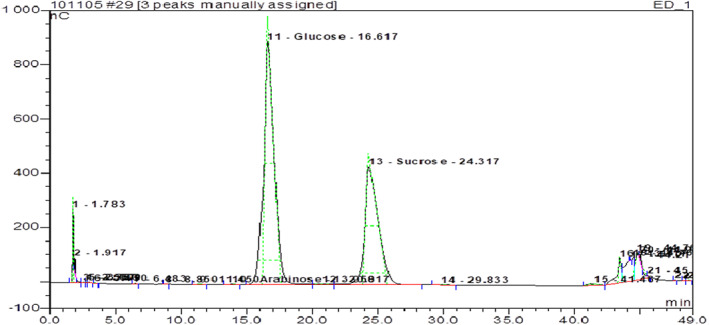
Essential sugars of five *Ziziphus jujuba* fruits using HPLC analysis (Chromatogram).

Chromatogram analysis showed three essential sugars classified in order of decreasing value: sucrose (24.3 min), glucose (16.6 min), and galactose (44.8 min) (Figure 4).

This composition varied deeply among provenances. In fact, Mahdia provenance was the richest one in glucose (136.51%) and sucrose (113.28%) contents (Table 2). The pulp's sugars were also rich in galactose ranging from 0.2% in Sfax to 0.45% in Mahdia.

**TABLE 2:** Sugar compositions of five *Ziziphus jujuba* fruits provenances using HPLC analysis.

	galactose	glucose	sucrose
mahdia	0.45 ± 0.00^a^	136.51 ± 1.18^a^	113.28 ± 2.56^a^
mahres	0.41 ± 0.01^b^	131.01 ± 0.7^b^	91.71 ± 1.13^b^
sfax	0.2 ± 0.00^d^	88.3 ± 1.97^c^	78.48 ± 2.64^c^
chotrana	0.36 ± 0.00^c^	115.4 ± 0.47^d^	91.08 ± 0.45^b^
rgueb	0.43 ± 0.02^a^	125.4 ± 0.17^b^	109.1 ± 0.27^d^

*Note*: The data are mean values of three measurements ± SE. For each column, values with the same letter indicate no‐significant differences at 5%.

Such results confirmed the high nutritional value of *Z. jujuba* fruit due to its richness in large quantities of nutrients and phytochemicals. So the idea to incorporate this fruit in some foods.

### 3.3 Total polyphenol, flavonoid and tannin contents

Many research projects focused on medicinal plants due to their huge role in both food and pharmaceutical industries. Therefore, a study of phenolic compounds has become a specific necessity due to their intervention to reduce the stress oxidative and to neutralize radicals in the human organism.

**FIGURE 5:**
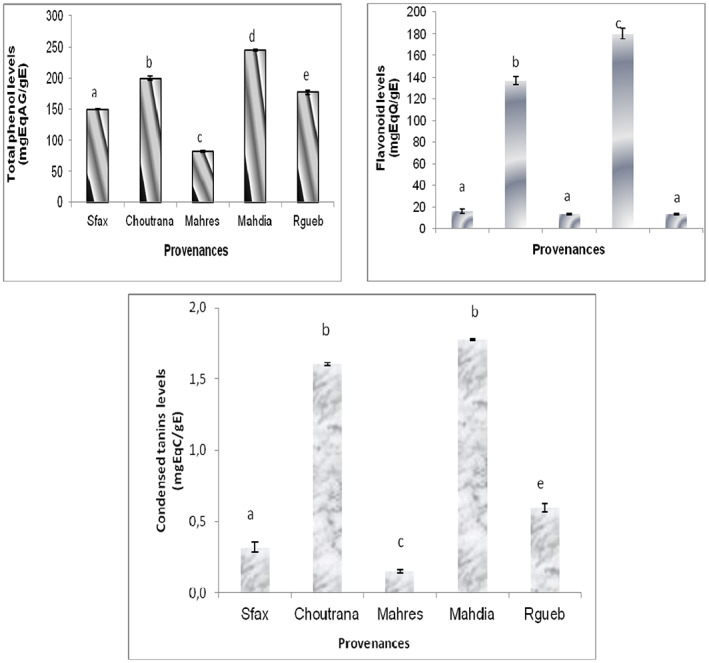
Phenolic compounds in fruits of five *Z. jujuba* provenances. The data are means values of three measurements. The confidence intervals were calculated at the threshold of 5%.

As shown in Figure 5, the richest provenance for phenolic compounds was Mahdia. The highest levels reached the values of 245.15 mg GAE/g DW, 180.23 mg QE/g DW, and 177 mg CE/g DW for total phenol, flavonoid, and tannins, respectively.

### 3.4 Biological activities

#### 3.4.1 Antioxidant activity

**FIGURE 6:**
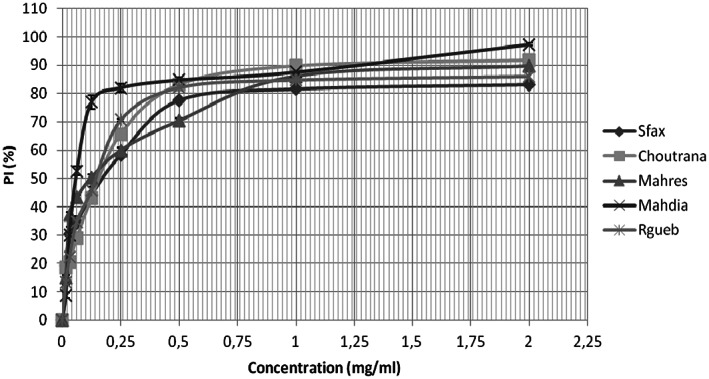
Variation of inhibition percentage (%IP) of five *Z. jujuba* provenances. The data are mean values of three measurements.

Results showed that antioxidant activity varied from not only one provenance to another but also among extracts. The inhibition levels increased with the increase of the extract concentrations. In fact, the inhibition yield heightened from 8.72% to 97.38% when extract concentration of Mahdia provenance moved from 0.016 to 2 mg/mL (Figure 6). At the concentration of 2 mg/mL, the percentage of inhibition reached the levels of 83.19%; 91.67%; 89.68%; 97.38% and 86.16% for Sfax, Choutrana, Mahres, Mahdia, and Regueb provenances, respectively.

The DPPH through the IC50 values (Concentration required for 50% inhibition) decreased slightly from 175 μg/mL (Sfax) to 55 μg/mL (Mahdia). Such results confirmed that the Mahdia provenance was the richest one and exceeded those obtained for the BHT (2,6‐di‐tert‐butyl‐4 methyl phenol) used as a standard (77.86 μg/mL).

#### 3.4.2 Antibacterial activities

Due to the multi‐resistance microorganisms, there were great demands to research new molecules from alternative sources like plants.

The antibacterial potential of various extracts revealed high differences between each of the tested provenances. The results indicated that the *S. aureus* was the most inhibited microorganism especially when they were treated by Sfax extracts. The diameters of zone inhibition expressed in mm varied from 12 to 20 mm (Table 3).

**TABLE 3:** Antibacterial activities of various extracts of five *Ziziphus jujuba* provenances.

Extraits[mg/mL] Souches	Sfax		Choutrana		Mahres		Mahdia		Regueb		Ampicilline	DMSO
	50	100	50	100	50	100	50	100	50	100	0.02	
*Staphylococcus aureus*	13	**20**	12	15	15	**17**	12	**16**	13	**18**	10	0
*Listeria monocytogenes*	10	13	12	15	10	14	11	14	10	15	16	0
*Kleibsella pneumoniae*	12	**18**	11	15	11	13	10	12	12	**16**	12.5	0
*Aeromonas hydrophila*	11	14	10	13	10	14	10	12	11	14	12.5	0

Abbreviation: DMSO: Dimethyl sulfoxide was used to calibrate the curve. Values indicated the diameters of zone inhibition expressed in mm. with extracts used at 25 mg/mL. The data are mean values of three measurements. Confidence intervals were calculated at the threshold of 5%. Ampicillin was used as standard. Extracts were used as concentration equal to 50 and 100 mg/ml.

In addition, the Sfax and Regueb provenances showed a strong antibacterial activity against *Kleibsella Pneumoniae* with an inhibitor diameter ranging respectively between 16 and 18 mm. The nutritional value of *Z. jujuba* fruits was deeply confirmed by the antioxidant and antibacterial activities. However, fresh fruits have a short shelf‐life. So it will be very necessary to transform it as a novel product in order to preserve it for a long time. So the idea to incorporate *Ziziphus* powder into cake.

### 3.5 Physical properties of cake supplemented by *Z. jujuba* powder

#### 3.5.1 Effects on water absorption

**FIGURE 7:**
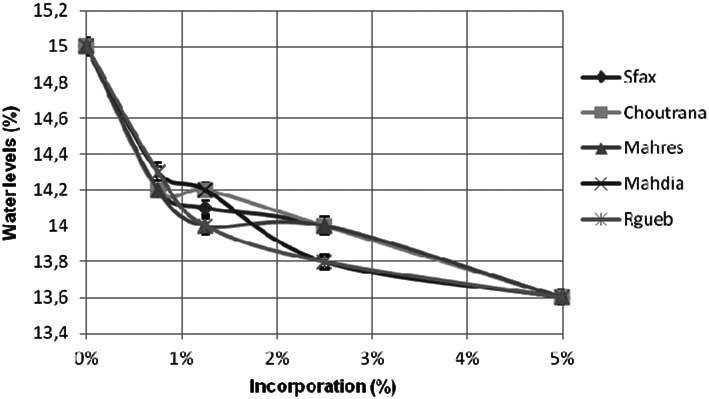
Water absorption (%) of cake supplemented by *Ziziphus jujuba* powders. The data are mean values of three measurements.

The water absorption levels, as shown in Figure 7, decreased from 15% (control cake) to 13.6% (5% of jujube incorporation). This diminution was more notable with the jujubes from Regueb provenance. Our results were compared to the Tunisian normative data (TN 51.21, 1989) that fixed the humidity of the flour between 13% and 16%.

As showed in Figure 7, the decline of the humidity influenced the yield of the final product (high‐quality flour) that could be conserved for a long period, without yeast or mold deterioration and food decomposition.

#### 3.5.2 Effects of ash contents

**FIGURE 8:**
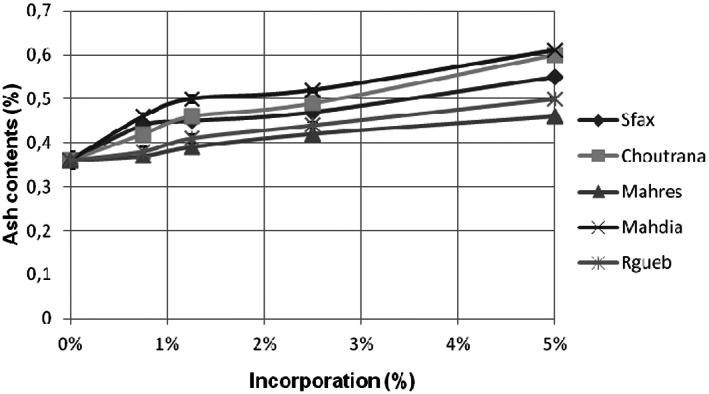
Ash contents (%) of cake supplemented by *Ziziphus jujuba* powder. The data are mean values of three measurements.

The ash contents informed us about the flour extraction (Figure 8). Our results showed that the ash levels increased with the augmentation of the jujube powder quantities incorporated. In order of increasing values, the richest provenance in ash contents was Mahdia with the value of 70%. This result could be explained by a low extraction rate (PS‐7) of this type of flour. Indeed, it was pastry flour derived from the central albumen of the wheat grain and devoid of the peripheral layers rich in minerals.

This study proved that edible and delicious *Z. jujuba* fruit could ameliorate some physical properties (the humidity and ash contents) of the cake formulated with jujube powder.

### 3.6 Rheologic properties of cake supplemented by *Z. jujuba* powder

Rheologic behavior briefly could predict dough processing parameters and the quality of the final product. Some rheological parameters, investigated in this study, were past tenacity, extensibility, swelling capacity, baking strength index, elasticity index, and falling number.

- Past tenacity

**FIGURE 9:**
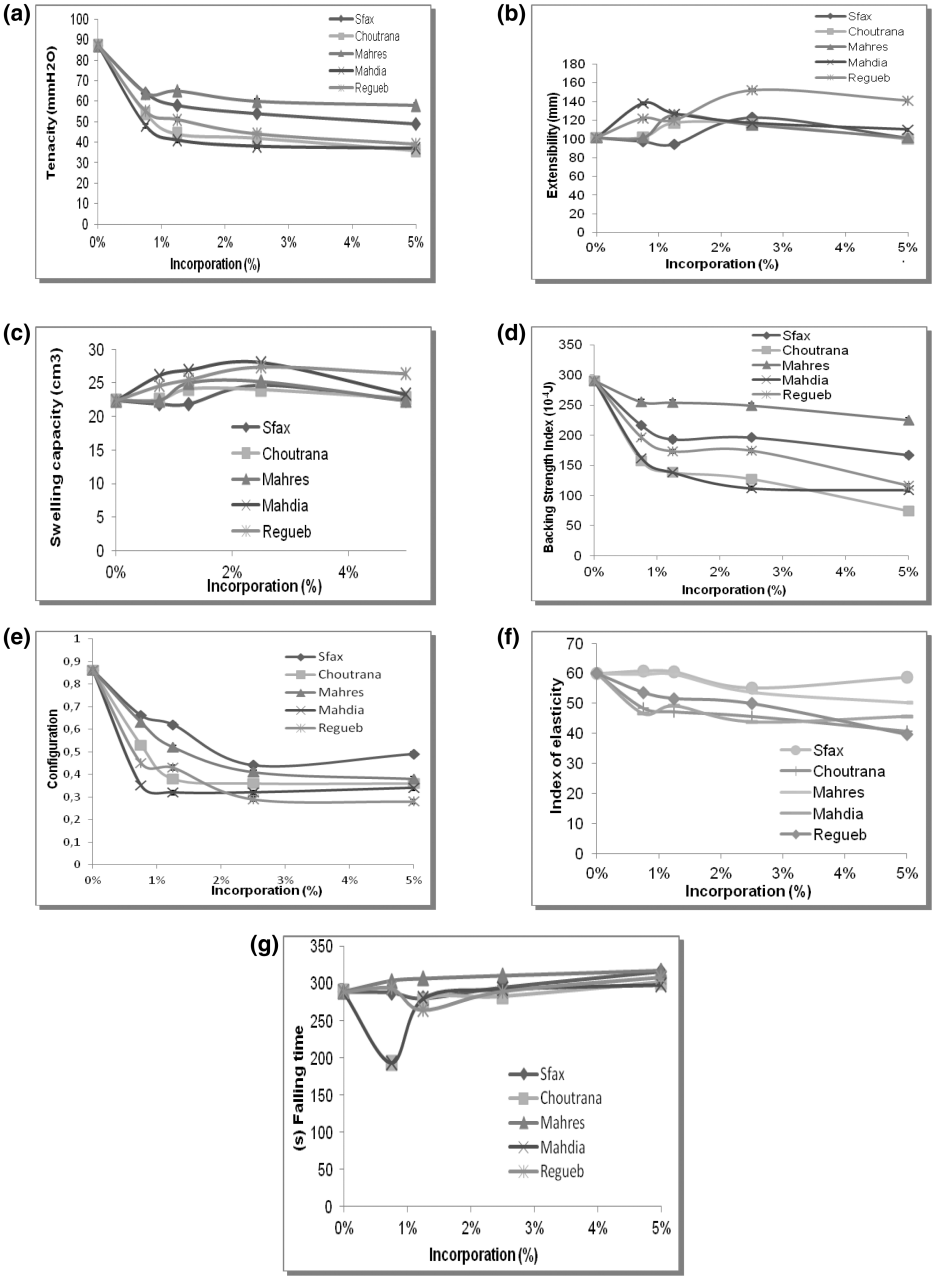
Rheologic properties of cake supplemented by *Ziziphus jujuba* powder. The data are mean values of three measurements.

Rheological properties of dough and gluten contents were greatly affected during the mixing process by the flour compositions (low or high protein contents), the processing parameters (mixing time, energy, and temperature) and the ingredients (water, salt, yeast, fats, and emulsifiers). As shown in Figure 9a, past tenacity deceased after the addition of the *Z. jujuba* powder, especially those of the Mahdia provenance powder. In fact, this parameter decreased from 87 to 37 mm H_2_O for the control and the *Z. jujuba* powder of Mahdia provenance, respectively.

- Extensibility

Contrarily, the incorporation of the *Z. jujuba* powder increased the extensibility of the past (Figure 9b). This augmentation was observed after adding the jujube powder at a rate of 3%, whereas, at certain levels of incorporation, this extensibility was reduced.

- Swelling capacity

This parameter informed us about dough extensibility. As shown in these results, we can conclude that the swelling capacity increased with the augmentation of the *Z. jujuba* powder, especially for the use of the Mahdia provenance powder (Figure 9 c).

- Baking strength index

The baking strength index was a protein quality parameter that expresses loaf volume. The results showed a decrease in baking strength with the increase in the rate of substitution of wheat flour by jujube powder (Figure 9d).

- Configuration

As shown in Figure 9 e, the incorporation of jujube powder at a rate of 5% decreased the configuration rate from 0.49% (for the control) to 0.28 (Rgueb provenance). This result confirmed that the dough was doubly extensible. So the elasticity of the dough increased with the incorporation of *Z. jujuba* powder.

- Index of elasticity

The elasticity of the jujube cake increased slightly by the addition of *Z. jujuba* powder from 59.9 (cake without *Z. jujuba* powder) to 58.7; 40.8; 50.2; 45.7 and 39.7 of Sfax, Choutrana, Mahres, Mahdia, and Regueb *Ziziphus* powder provenances, respectively added at levels of 5% (Figure 9 f).

- Falling time

The falling time increased with the rate of *Ziziphus* powder addition without exceeding the Tunisian norm (Figure 9 g). In addition, the increase in falling number demonstrated a decrease in α‐amylase activity that created sticky dough.

#### 3.6.1 Effects on wet, index, and dry gluten

The protein dough strength measurements and gluten properties, in particular, significantly impacted dough strength measurements. In fact, fruits with high gluten levels could ameliorate dough handling properties. The rheological properties of the gluten (the combination of its viscous, elastic, and cohesive properties) were also studied….

**FIGURE 10:**
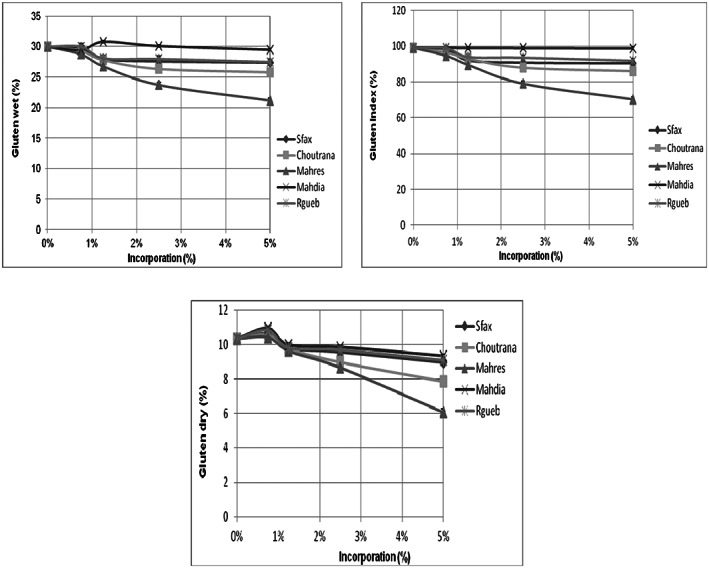
Effects of *Ziziphus jujuba* powder on wet, index and dry gluten of dough The data are mean values of three measurements.

Results mentioned that the incorporation of *Z. jujuba* powders reduced the wet, index, and dry gluten contents of the pastry flour. This reduction was observed for Mahres provenance (Figure 10).

In fact, based on ISO 5531 and 6645 norms, the wet gluten levels varied between 24 and 30%. That was compared to our sample (25% and 30%). This characteristic could inform us about the quality of the flour and its rheological parameter. As showed in this study, the incorporation of *Ziziphus* powder did not modify the index gluten of dough. In fact, this level was between 86% and 98% that was compared with the control test.

In fact, higher amounts of gluten proteins increased the dough elasticity and prevent hamper molding of the dough (Hanee, 2013). In addition, the gluten index allowed us to highlight the deterioration caused by insects and heat. At 5% of *Z. jujuba* incorporation, dry gluten percentages ranged between 6% and 9.4%. This inveterate the conservation of heat‐resistant protein, so our flours keep their nutritional qualities.

According to this result we can validate a novel method to conserve *Z. jujuba* fruits and avoid their soilage for a long period.

### 3.7 Sensory analysis

**FIGURE 11:**
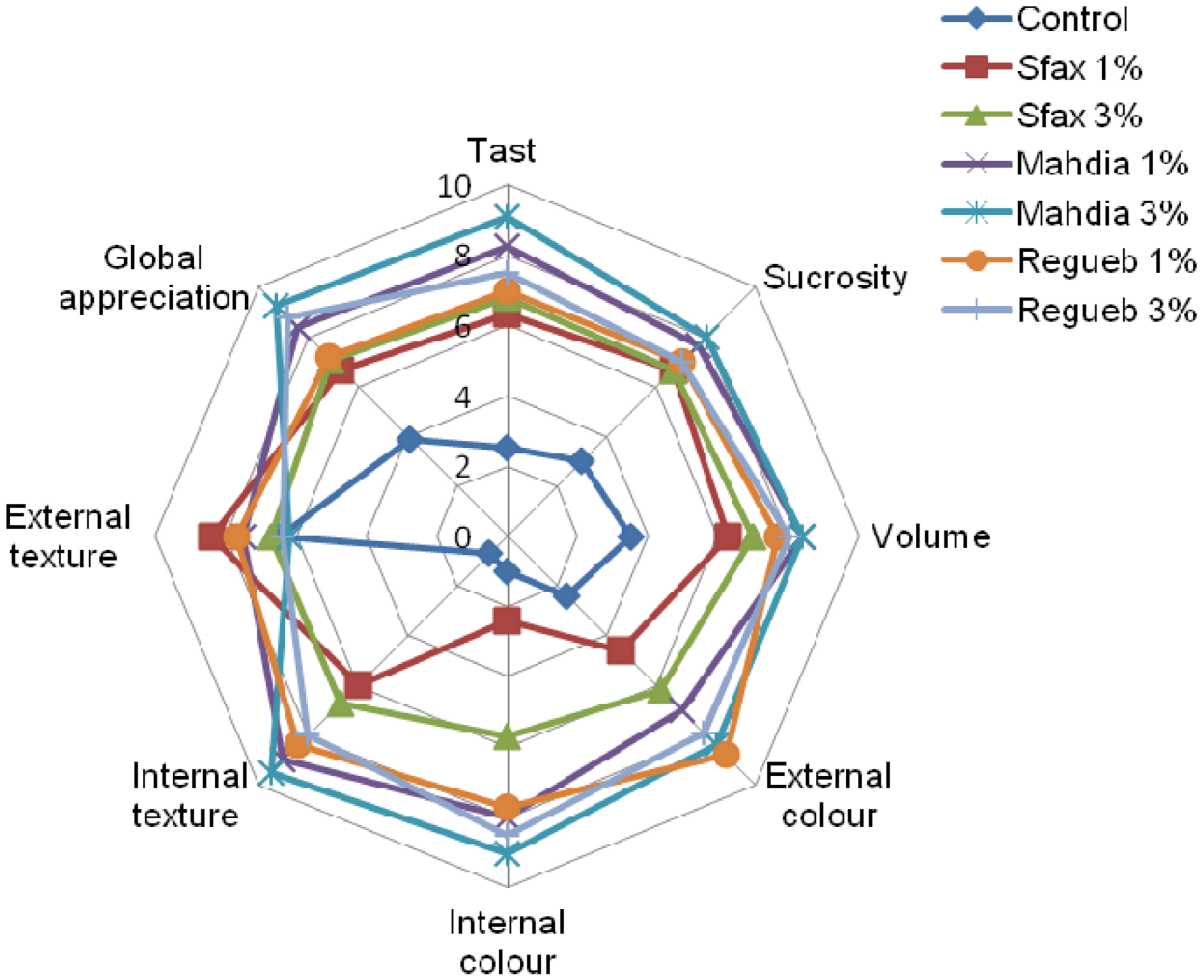
Sensory analysis of cake supplemented by *Ziziphus jujuba* powder. The data are mean values of three measurements.

The result of sensory analysis of *Z. jujuba* powder cake was evaluated through taste, color, texture, volume, and global appreciation (Figure 11). Results showed that consumer scores were increased with the increasing supplementation level. In fact, highest scores were attributed to the cake supplied with 3% jujube powder.

#### 3.7.1 Taste of cake

Sensory analysis of cake supplemented by *Z. jujuba* powder showed a great change in the taste of the cake. This variation between provenances could be attributed to the richness of Z. powder on lipoxygenase inhibitory activity, so the synthesis of many volatile compounds such as alcohols, ketones, and aldehydes (Molteberg et al., 1995).

Taste scores were affected by the jujube powder substitution. From the mean scores of the organoleptic evaluation, the Mahdia provenance powder supplemented at the concentrations of 1% and 3% were higher for all examined attributes and ranked at 9.1. Oppositely, consumer scores increased with increasing supplementation levels. This characteristic could be attributed to the strong lipase activity that caused many oxidative reactions responsible for the savory taste (O'Connor et al., 1992).

This incorporation modified the sucrosity of cake and reached the maximum values (7.7 and 8) for Mahdia provenance added at rates of 1% and 3%, respectively. Such variability could be attributed to high levels of sugar fruits.

#### 3.7.2 Color

The color of the cake expressed the sensation the cake produced on the eyes. The difference between the mean of the provenance was quite significant. In fact, the comparison between provenances showed that the color of Mahdia provenance had the highest mean (11). The Sfax provenance had a low mean relative to the other provenances (2.4).

#### 3.7.3 Texture

The texture of the cake referred to the smoothness or appearance of the feel of the cake in the mouth. This variation changed with the rate of jujube powder incorporation. In fact, compared to the control, in which the texture was heavy estimated (0.75), the texture levels ranged between 6.9 and 9 for Sfax and Mahdia provenances, respectively, incorporated rates of 1% and 3%. The result indicates that the texture of the Mahdia provenance was preferred to other samples. In addition, as shown in Figure 11, this parameter increased with the addition of *Z. jujuba* powder. This increase could be explained by the high content of polysaccharides, which may lead to the dilution of gluten and extensive gluten structure (Zarroug et al., 2021).

#### 3.7.4 Volume

The difference in volume of all samples was easily detected among consumers. However, the volume of the control cake was low with the mean scores of the cakes evaluated equal to 4. This could be explained by the absence of cells in this cake.

The comparison between provenances showed that the cake enriched with Mahdia jujube powder (1% et 3%) had scored the highest volume mean (8.4) by panelists, while cake sample supplemented with Sfax jujube powder at 1% and 3% were less accepted than all other provenances, but more important than those made only with wheat flour (3.5). Such variability in volume varied not only according to the provenances but also the rates of their additions to the cakes.

#### 3.7.5 Global appreciation

This parameter expresses how the panelists accept the jujube cake. As shown in Figure 11, consumers were more inclined to accept cakes of Mahdia and Regueb provenances with the means of 9.5 and 8.5, respectively. In addition, cake jujube supplemented with Sfax powder (1%) were less accepted.

## 4 DISCUSSION

*Ziziphus jujuba* fruit characterizations showed that protein contents varied between provenances and attempt the value of 0.60%. Such results were higher than those cited by El Aloui (2013) that obtained levels varying from 0.01% to 0.02%. Other research showed that the protein levels decreased with the maturity of the fruit (Moradinezhad et al., 2016) from 3.34% (full maturity) to 6.35% (immature fruit). So we could conclude that our fruits were at full maturity.

The richness on carbohydrate contents proved the high caloric levels of Mahdia provenance that exceeds the value of 181.39 Kcal/g FW compared to Sfax provenance whose caloric levels did not exceed 135.54 Kcal/g.

In addition, *Z. jujuba* fruits exhibited a valuable richness on the humidity contents especially these of Mahdia provenance. This variability could be due to some conditions such as climatic conditions (temperature, precipitation…), geographic location… This low percentage facilitated the storage of *Z. jujuba* fruits. Our results were in comparison to other species such as Z. *mauritiana* and Z. *spina‐christi* with levels of 46% and 85%, respectively (Anthony, 2005).

Total sugar levels ranged between 14.12% to 16.59%. Such levels were found to be lower (59.87%) than those reported in *Z. lotus* fruits by Masmoudi et al. (2021). Results obtained in this study by HPLC analysis were contradicted with these reported by Zhang et al. (2020) who confirmed that sucrose (40.3%) and mannose (21%) were the prominent sugars present in *jujuba* fruits. The highest content of all the soluble sugars was recorded at the full‐red stage.

Secondary metabolites analysis enhanced the richness of our extracts on phenolic compounds. Levels were higher than those found (10.43 to 15.85 mg/ L) by Elaloui et al. (2014). Other works done by Rashwan et al. (2020) showed that total phenolic ranged from 1.1 to 2.4 g/100 g DW, and flavonoid contents ranged from 0.7 to 1.8 g/100 g DW. These results could validate jujube fruit as a good source of phenolics compound and therefore recommended it by nutritionists to be part of our diet. This idea was also confirmed by Rashwan et al. (2020).

The cake supplemented by *Z. jujuba* powder increased the ash contents to 70% especially for the Mahdia provenance. These results were not in accordance with the findings of John et al. (2021) who noticed that ash contents increased with increasing levels of *Finger millet* flour. They explained that the high level of the ash content in the composite cakes was due to the richness of *Finger millet* flour on minerals.

In order to decrease the consummation of our specie, the *Z. jujuba* fruits were incorporated into Neapolitan pizza, the sensory analysis proved that this incorporation increased the fiber, total phenolic and flavonoid contents, and the radical scavenging activity (Falciano et al., 2022).

In addition, the *Z. jujuba* fruits incorporation decreased the tenacity of our cake. This could be attributed to the richness of our fruits on proteins without glutelin (protein responsible for past tenacity) or also for the diluting gluten (Godon et Loisel, 1984). The extensibility was also reduced after this incorporation. This decline could be attributed to the diminution in the gluten level in particular the gliadins level that gave rise to larger cake volume during baking. Such variation reflected an increase in the gas retention property within the dough bubble and thus the retention of carbon dioxide within the cake dough. From these results, the dough which will be obtained from the basis of the mixture of two flours (wheat and *Z. jujuba*) will exhibit a decreasing density with the rate of incorporation and a cake of increasing volume.

In addition, the increase in falling number demonstrated a decrease in α‐amylase activity that created sticky dough. This idea was also cited by Tozatti et al. (2020). In opposition, the diminution of amylasic activities decreased the fermentation of the dough.

Our results showed that the incorporation of *Z. jujuba* powder ameliorates the physicochemical and rheological characteristics of the dough by reducing its humidity; gluten yields. So *Z. jujuba* powder could be recommended by nutritionists and industrial to formulate cake supplement with *Z. jujuba* powder. According to overall acceptability of results, the cake enriched with Mahdia jujube powders used at dose of 3% and 1% were the most acceptable, by panelists. Other studies carried out on biscuits supplemented with jujube flour had as acceptable quality as the control (Masmoudi et al., 2021). In addition, a diminution in acceptability of cookies at higher level of substitution with *Ziziphus lotus* was observed by Zarroug et al. (2021).

## 5 CONCLUSION

This study proved the benefits of *Z. jujuba* fruits as an active ingredient in the food industry by transforming it to cake. In fact, the *Z. jujuba* fruits incorporation increased the physicochemical and rheological characteristics (humidity, gluten yields, tenacity, falling time, and configuration) of the dough. In addition, cakes prepared with 3% (w/w) fruit powders of Mahdia provenance had comparatively the best nutritional and sensory characteristics over other provenances. According to these results we can validate a novel method to conserve *Z. jujuba* fruits especially these of Mahdia provenance and avoid their soilages for a long period. *Ziziphus* fruit was advised to be to be part of our diet.

## CONFLICT OF INTEREST STATEMENT

No potential conflict of interest was reported by the author.

## Data Availability

Research data are not shared

## References

[fsn33276-bib-0001] Anjum, M. A. , Haram, A. , Ahmad, R. , & Bashir, M. A. (2020). Physico‐chemical attributes of fresh and dried indian jujube (*Zizyphus mauritiana*) fruits. Pakistan Journal of Agricultural Sciences, 57(1), 165–176.

[fsn33276-bib-0027] Anthony, C. (2005). Areview of Ziziphus spina‐christi Technical (Ed.) 3P India.

[fsn33276-bib-0002] Audigie, L. , Figarella, J. , & Zonszain, F. (1978). Manipulation biochimique (pp. 27–74). Doin. Paris.

[fsn33276-bib-0003] Ben Hmed, M. , Rigane, G. , Ben Salem, R. , Zouari, N. , & Cherif, S. (2020). Phytochemical and antioxydant activities of *Schinus molle* L. extract. Revue Roumaine de Chimie, 65, 173–178. 10.33224/rrch/2020.65.2.06

[fsn33276-bib-0004] Dharajiy, D. , Shah, M. , & Bajpai, B. (2016). Biosorption of acid black 52, an azo dye from aqueous solution using pre‐treated biomass of *aspergillus fumigatus* A23. Pollution Research, 36, 667–676.

[fsn33276-bib-0005] Drake, M. A. , Drake, S. , Bodyfelt, F. , Clark, S. , & Costello, M. (2004). The sensory evaluation of dairy products (pp. 1–6). Springer Science & Business Media.

[fsn33276-bib-0006] Earp, C. F. , Akingbala, J. O. , Ring, S. H. , & Rooney, L. W. (1981). Evaluation of several methods to determine tannins in sorghums with varying kernel characteristics. Cereal Chemistry, 58, 234–283.

[fsn33276-bib-0030] El Aloui, M. (2013). Suivi de la phénologie et caractérisation morpho‐chimique comparés de quatre écotypes de Ziziphus jujuba (Miller) dans la station expérimentale de Rouhia (Tunisie) (semi‐aride supérieur). Thèse de doctorat, INAT, 172p.

[fsn33276-bib-0007] Elaloui, M. , Ghazghazi, H. , Ennajah, A. , Manaa, S. , Guezmir, W. , Karray, N. B. , & Laamouri, A. (2016). Phenolic profile, antioxidant capacity of five *Ziziphus spina‐christi* (L.) Willd. Provenances and their allelopathic effects on *Trigonella foenum‐graecum* L. and *Lens culinaris* L. seeds. Natural Product Research, 31, 1209–1213. 10.1080/14786419.2016.1226830 27618365

[fsn33276-bib-0008] Elaloui, M. , Haouel, S. , Ben Nasr, R. , Ghazghazi, H. , Emna, B. , Ammari, Y. , Ben Jemâa, J. M. , & Laamouri, A. (2020). Characterization of epicathechin contents in the *Ziziphus spina‐christi* L. root extracts using LC‐MS analyses and their insecticidal potential. Plant Biosystems, 155 (2), 1–9. 10.1080/11263504.2020.1779837

[fsn33276-bib-0009] Elaloui, M. , Laamouri, A. , Albouchi, A. , Cerny, M. , Mathieu, C. , Vilarem, G. , & Hasnaoui, B. (2014). Chemical compositions of the Tunisian *Ziziphus jujuba* oil. Emirates Journal of Food Agriculture, 26, 602–608. 10.9755/ejfa.v26i7.17513

[fsn33276-bib-0010] Essghaier, B. , Dhieb, C. , Rebib, H. , Ayari, S. , Rezgui, A. , Boudabous, A. , & Sadfi‐Zouaoui, N. (2014). Antimicrobial behavior of intracellular proteins from two moderately halophilic bacteria: Strain J31 of *Terribacillus halophilus* and strain M3–23 of *Virgibacillus marismortui* . Journal of Plant Patholology and Microbiology, 5, 5–1. 10.4172/2157-7471.1000214

[fsn33276-bib-0011] Etebari, M. , Zolfaghari, B. , Jafarian‐Dehkordi, A. , & Mirzaei, A. (2015). Hydroalcoholic and polyphenolic extracts of *Ziziphus jujuba* mill. Fruits prevent methyl methane sulfonate‐induced DNA damage in HepG2 cells. Pharmaceutical and Biomedical Research, 1, 20–30. 10.18869/acadpub.pbr.1.3.20

[fsn33276-bib-0012] Falciano, A. , Sorrentino, A. , & Di Pierro, P. (2022). Development of functional Pizza Base enriched with jujube (*Ziziphus jujuba*). Powder Foods, 11 (10), 458. 10.3390/foods11101458 PMC9141078 35627028

[fsn33276-bib-0013] Hanee, M. A. (2013). Cake flour: Functionality and quality. European Scientific Journal, 9(3), 166–180.

[fsn33276-bib-0014] John, A. , Bigson, K. , Maureen, N. A. , & Dorothy, A. (2021). Quality characteristics and sensory evaluation of cakes produced from composite blends of wheat (*Titricum Aestivum* L.) and finger millet (*Pennisetum Glaucum*) flour Eurasian. Journal of Food Science and Technology, 5(2), 190–204.

[fsn33276-bib-0015] Khemiri, L. , Larsson, H. , Kuja‐Halkola, R. , D'Onofrio, B. M. , Lichtenstein, P. , Jayaram‐Lindstrom, M. N. , & Latvala, A. (2020). Association of parental sub‐stance use disorder on offspring cognition: A population family‐based study. Addiction, 115, 326–336. 10.1111/add.14813 31503371

[fsn33276-bib-0029] Kjeldahl, J. (1883). A new method for the determination of nitrogen in organic matter. Zeitschrift für Analytische Chemie, 22, 366–382. 10.1007/BF01338151

[fsn33276-bib-0016] Mahdhi, A. , Ghazghazi, H. , El Aloui, M. , Ben Salem, R. , & Rigane, G. (2021). Phenolic and fatty acid profiles in *Pinus halepensis* mill. Seeds by LCESI‐MS and GC: Effect of drying methods on chemical composition. Food Science and Nutrition, 25, 1907–1916. 10.1002/fsn3.2151 PMC8020912 33841809

[fsn33276-bib-0017] Masmoudi, M. , Yaich, H. , Borchani, M. , Mbarki, R. , & Attia, H. (2021). Chemical, physical and sensory characteristics of biscuits enriched with jujube (*Zizyphus lotus* L.) flour and fiber concentrate. Journal of Food Science Technology, 58(4), 1411–1419. 10.1007/s13197-020-04652-7 33746269 PMC7925756

[fsn33276-bib-0018] Molteberg, E. L. , Vogt, G. , Nilsson, A. , & Frolich, W. (1995). Effects of storage and heat processing on the content and composition of free fatty acids in oats. Cereal Chemistry, 72, 88–93 D: 42430844.

[fsn33276-bib-0031] Moradinezhad, F. , Setayesh, F. , Mahmoodi, S. , Khayyat, M. (2016). Physicochemical properties and nutritional value of jujube (Ziziphus jujuba Mill.) fruit at different maturity and ripening stages. International Journal of Horticultural Science and Technology, 3(1), 43–50.

[fsn33276-bib-0019] Najjaa, H. , Ben Arfa, A. , Elfalleh, W. , Zouari, N. , & Neffati, M. (2020). Jujube (*Zizyphus lotus* L.): Benefits and its effects on functional and sensory properties of sponge cake. PLoS One, 15, 1–14. 10.1371/journal.pone.0227996 PMC7034905 32084133

[fsn33276-bib-0020] O'Connor, J. , Perry, H. J. , & Harwood, J. L. (1992). A comparison of lipase activity in various cereal grains. Journal of Cereal Science, 16, 153–163. 10.1016/S0733-5210(09)80147-1

[fsn33276-bib-0021] Rashwan, A. K. , Karima, N. , Shishira, M. R. I. , Baoa, T. , Lua, Y. , & Chena, W. (2020). Jujube fruit: A potential nutritious fruit for the development of functional food products. Journal of Function Foods, 75, 104205. 10.1016/j.jff.2020.104205

[fsn33276-bib-0022] Singleton, V. L. , & Rossi, J. A. (1965). Colorimetry of total phenolics with phosphomolybdic‐phosphotungstic acid reagents. American Journal of Enology and Viticulture, 16, 144–158.

[fsn33276-bib-0023] Sun, B. , Richardo‐Da‐Silvia, J. M. , & Spranger, I. (1998). Critical factors of vanillin assay for catechin and proanthocyanidins. Journal of Agriculture Food Chemestry, 46 (10), 4267–4274. 10.1021/jf980366j

[fsn33276-bib-0024] Tozatti, P. , Güldiken, B. , Fleitas, M. C. , Chibbar, R. N. , Hucl, P. , & Nickerson, M. T. (2020). The interrelationships between wheat quality, composition, and dough rheology for a range of Western Canadian wheat cultivars.Cereal. Chemistry, 97, 1010–1025.

[fsn33276-bib-0025] Zarroug, Y. , Sriti, J. , Sfayhi, D. , Slimi, B. , Alloucha, W. , Zayani, K. , Hammami, K. , Sowalhia, M. , & Kharrat, M. (2021). Effect of addition of Tunisian *Zizyphus lotus* L. fruits on nutritional and sensory qualities of cookie. Italian Journal of Food Science, 33(4), 84–97.

[fsn33276-bib-0026] Zhang, Q. , Wang, L. , Wang, Z. , Liu, Z. , Zhao, Z. , Zhou, G. , Liu, M. , & Liu, P. (2020). Variations of the nutritional composition of jujube fruit (*Ziziphus jujuba* mill.) during maturation stages. Intnational Journal of Food Propriety, 23 (1), 1066–1081. 10.1080/10942912.2020.1770281

